# The Role of B1 Cells in Systemic Lupus Erythematosus

**DOI:** 10.3389/fimmu.2022.814857

**Published:** 2022-03-28

**Authors:** Zhou She, Cuifang Li, Feifeng Wu, Jueyi Mao, Min Xie, Marady Hun, Amin Sheikh Abdirahman, Senlin Luo, Wuqing Wan, Jidong Tian, Chuan Wen

**Affiliations:** Department of Pediatrics, The Second Xiangya Hospital, Central South University, Changsha, China

**Keywords:** mice, human, systemic lupus erythematosus (SLE), B1 cells, antibody

## Abstract

Systemic lupus erythematosus (SLE) is a systemic autoimmune disease characterized by multisystemic and multi-organ involvement, recurrent relapses and remissions, and the presence of large amounts of autoantibodies in the body as the main clinical features. The mechanisms involved in this disease are complex and remain poorly understood; however, they are generally believed to be related to genetic susceptibility factors, external stimulation of the body’s immune dysfunction, and impaired immune regulation. The main immune disorders include the imbalance of T lymphocyte subsets, hyperfunction of B cells, production of large amounts of autoantibodies, and further deposition of immune complexes, which result in tissue damage. Among these, B cells play a major role as antibody-producing cells and have been studied extensively. B1 cells are a group of important innate-like immune cells, which participate in various innate and autoimmune processes. Yet the role of B1 cells in SLE remains unclear. In this review, we focus on the mechanism of B1 cells in SLE to provide new directions to explore the pathogenesis and treatment modalities of SLE.

## Introduction

Systemic lupus erythematosus (SLE) is a chronic autoimmune disease with an incidence of approximately 0.3–241/100,000. It is characterized by abnormal activation of the immune system, overactivation of T and B cells, production of a large number of autoantibodies that bind to self-tissues to form immune complexes and deposits, and damage to multiple organ systems throughout the body; the condition has a poor prognosis (1–3). B cells are antibody-producing cells that play a crucial role in the development of SLE and have been studied extensively. B-cell antagonists, such as rituximab and belimumab, are effective in the treatment of SLE; however, therapeutic outcomes are inadequate, which may be related to the neglected B1 cell population. B1 cells are a group of B cells that are colonized in the peritoneum and pleural cavities, which enables them to evade drugs. Moreover, the growth and development of B1 cells do not depend on the B-cell activation factor; thus, drugs that target B cells are ineffective (4–8).

**Figure 1:**
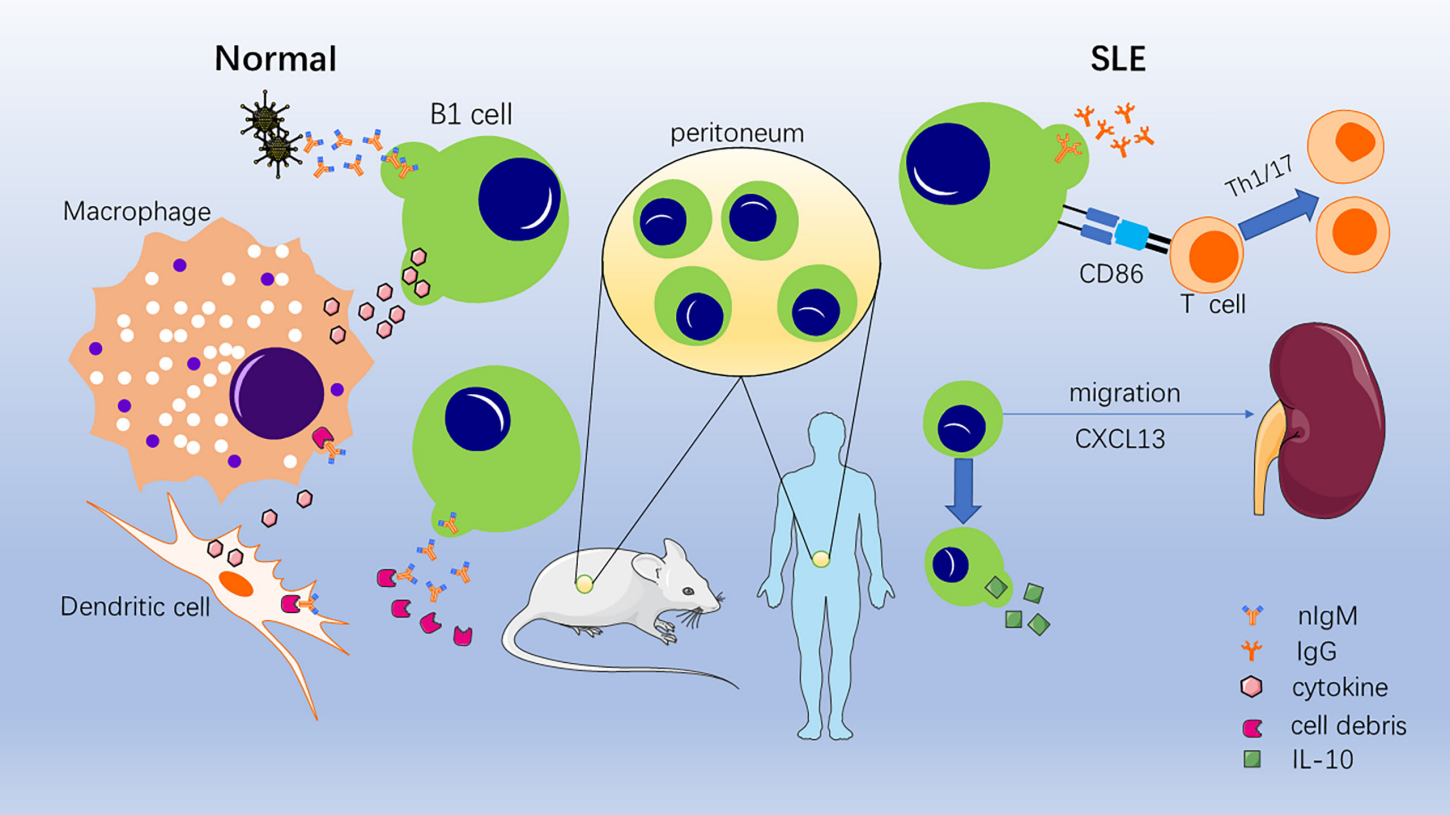
Role of B1 cells under normal conditions and systemic lupus erythematosus (SLE) conditions. In normal situations, B1 cells secrete nIgM binding invading pathogenic bacteria and necrotic and apoptotic cellular components. Then, phagocytosis and clearance are accelerated, and cytokines are secreted to activate macrophages and dendritic cells. In SLE conditions, B1 cells convert to secrete self-reactive IgG; promote the conversion of T cells to Th1/17 cells; migrate to the kidney, thymus, and spleen; and secrete IL-10.

B cells are divided into three categories according to the order of their appearance during fetal development: B1 cells, B2 cells, and marginal zone (MZ) B cells. B2 cells, which are usually referred to as follicular B cells, are produced by bone marrow hematopoietic stem cells and subsequently mature in secondary lymphoid organs, where they participate in adaptive immunity by producing memory cells and antibodies. MZ B cells are a population of B cells located between the splenic red and white pulp that secrete IgM to participate in immunity (9–11). In contrast, B1 cells are a population of B cells that primarily arises during the fetal stage, colonizes the peritoneal cavity, and is capable of self-renewal and spontaneous IgM secretion. Initially, a population of B cells expressing CD5 molecules, now called B1a cells, was found in mice capable of spontaneous IgM secretion, and their development, phenotype, tissue distribution, and functional characteristics differed from B2 cells (11, 12). A population of B1 cells with a similar functional profile, but one that does not express CD5 molecules, was subsequently identified, which is known as B1b cells (13, 14). It is generally believed that B1b cells have a broader antigen repertoire and play a role in long-term humoral memory, known to be important in resolving *Borrelia* and other infections (15–19). Because the distinction between B1a and B1b cells is not entirely clear, we will refer to them as B1 cells. Under normal conditions, B1 cells primarily act as a bridge between intrinsic and adaptive immunity by spontaneously secreting multi-reactive IgM, clearing pathogenic bacteria at an early stage, and activating macrophages and dendritic cells by combining their own necrotic and apoptotic components to promote clearance and secrete cytokines (7, 11, 20). It has since been demonstrated that B1 cells are involved in numerous autoimmune diseases, such as rheumatoid arthritis, immune hemolytic anemia, and SLE (7, 13, 21, 22). Moreover, in some autoimmune disease models, such as SLE, diabetes, and autoimmune hemolytic anemia, B1 cells have been activated and proliferated (23–26). In addition, B1 cells have been linked to autoimmune diseases in a variety of gene-edited mice. In galectin-9-deficient mice, B1 cells are activated readily and expanded with autoantibodies, which drive autoimmune responses, such as spontaneous germinal center formation and nephritis (27). Generation and activation of self-reactive B1 cells have been found in cytotoxic T lymphocyte-associated antigen (CTLA-4)-deficient mice and SPA-1-deficient mice (28, 29). Therefore, this raises the question: what role do B1 cells play in SLE (**Figure 1**)?

## Role of B1 Cells in Systemic Lupus Erythematosus Mice

B1 cells in mice, mainly with the CD5 molecule, are their marker. As early as the 1990s, numerous scholars found that clearance of B1 cells, could, to some extent, alleviate SLE, as observed in NZB/NZW F1 mice and NZM2410 lupus-prone mice (21, 30–33). Furthermore, a rise in the number of B1 cells has been found in various mouse models of SLE (8, 34). It has been found that the gene Cdkn2c, a gene encoding for cyclin-dependent kinase inhibitor p18^Ink4c^, which can regulate the cell cycle to control B-cell multiplication, is associated with the expansion of B1 cells and the development of glomerulonephritis (35–37). Therefore, how exactly do B1 cells contribute to the development and progression of SLE?

### Antibody Secretion

Normally, as intrinsic immune cells, B1 cells do not require foreign antigen stimulation and spontaneously secrete large amounts of low-affinity, multi-reactive natural IgM (nIgM) in a T cell-independent manner. These nIgM bind to debris and recruit C1q, which activates the classical complementing pathway and promotes phagocytes (7, 38). However, it has been found that in addition to nIgM, B1 cells produce rheumatoid factor and immunoglobulin G (IgG) antibodies against single-stranded DNA (anti-ssDNA) (39, 40). Subsequent studies found that a class of B1 cells expressing programmed death-ligand 2, stimulated by interleukin (IL)-21, produces high-affinity anti-double-stranded DNA (anti-dsDNA) antibodies belonging to IgG1 and IgG2b types (39). Moreover, *in vitro* experiments have found that natural killer T cells assist B1 cells in producing IgG2a-type anti-dsDNA, which is an antibody that binds primarily to the glomerular basement membrane and causes glomerular damage (41, 42).

The generation of such high-affinity IgG antibodies that bind to its own tissues as described above may be associated with an increased expression of recombination activating genes (RAG) by B1 cells in SLE mice, and the number of B1 cells with high RAG expression has been shown to correlate with disease activity (43, 44). Normal B cells undergo recombination binding when developing in the bone marrow. Further receptor editing clears self-reactive cells, which results in immune tolerance, an important self-protective process (44–46). Normally, such B cells with high RAG expression are only found in the bone marrow and secondary lymphoid organs. However, when their antigen recognition profile changes, they become potentially auto-reactive antibody-producing cells. In SLE mice, the threshold for B1 cells to undergo rearrangement is reduced, and they exhibit frequent expression of RAG; moreover, the presence of a large number of uncleared autoantigenic stimuli makes B1 cells highly susceptible to switching, producing autoantibodies with high affinity, damaging tissue, and contributing to the development and progression of the disease.

### The Stimulation of CD4+ T Cells

Although B1 cells produce antibodies in a T cell-independent manner, they can act as antigen-presenting cells and stimulate the proliferation, activation, and differentiation of T cells. With the help of CD80/86 expression, B1 cells can stimulate CD4+ T-cell activation *in vitro* and preferentially differentiate towards Th1 and Th17 cells (13). It has been found that B1 cells primarily induce T cells to secrete γ-interferon and IL-17, which reflects differentiation to Th1 and Th17 cells, and their induction ability is much greater than that of B2 and dendritic cells (47). Similar results were also observed in SLE-prone mice, and subsequent studies further demonstrated that CD86 is the primary costimulatory molecule, with CD80 playing a synergistic role, and that cytokines, such as IL-2/10, inhibit this process (26, 48). As a proinflammatory phenotype, Th1/17 cells can produce cytokines, such as γ-interferon and IL-17, which play a role in SLE (49–51). By stimulating the proliferation of CD4+ T cells and differentiation towards Th1/17 cells, B1 cells have a promotive effect on SLE.

### Migration to Specific Target Organs

B1 cells predominantly accumulate in the peritoneum and pleural cavities, where they self-renew and produce natural antibodies that exert immune effects. Therefore, under normal conditions, B1 cells have difficulty encountering antigens and other immune cells *in vivo* and do not produce an autoimmune response (7, 8, 26, 52, 53). However, in SLE model mice, the abnormal distribution of multiple B lymphocyte chemoattractants (BLC) causes B1 cells to accumulate in various inflammatory regions, which damages target organs (8). Prior to the development of nephritis in BWF1 lupus mice, *in vivo* BLC expression was increased in the thymus, lungs, and kidneys and decreased in the peritoneal cavity for homing. B1 cells were more attracted to these BLC than to B2 cells, and thus, they aggregated in and infiltrated these target organs (53). Subsequent studies revealed that the perivascular space of the thymus is enlarged in BWF1 mice, and the thymic vascular endothelial cells increase the expression of BLC and adhesion factors, attracting B1 cells expressing CD11b, which stimulates thymic T-cell activation and proliferation in the presence of IL-2 (54). CXC chemokine ligand 13 (CXCL13) is an important chemokine that serves as a marker of SLE severity. Through the proteoglycan biglycan-toll-like receptor (TLR)2/4 pathway, CXCL13 levels can be increased in the kidney, which aggregates B1 cells that interact with other cells and damage the glomerular and tubular interstitium (55), whereas, through TLRs, the aggregation of B1 cells in the spleen can be regulated (52). Additionally, some of the B1 cells that aggregate in the target organ may be cells that produce IgG antibodies after the occurrence of class switching (56). However, it has been noted that B1 cells that aggregate in the renal mesenchyme do not produce antibodies; rather, they rely primarily on communication with other cells to induce an effect (34). By migrating to the inflamed organ, B1 cells that were originally confined to the peritoneal cavity are able to communicate with numerous other immune cells, exert their antigen-presenting cell capacity, stimulate the proliferation and differentiation of T cells, and switch to a proinflammatory phenotype. However, they are also exposed to more *in vivo* antigens, which may stimulate class switching, produce high-affinity auto-IgG, or release more multi-reactive IgM, which binds to tissue and exposes further autoantigens.

### Secretion of Cytokines

B1 cells are a type of immune cell that can secrete cytokines that regulate immune function, such as interferon-γ, IL-6/10/12, and tumor necrosis factor α. Among these cytokines, the most well studied is IL-10 (8, 57–61). IL-10 plays a role in many autoimmune diseases, including SLE. On the one hand, IL-10 inhibits the production of inflammatory factors, the function of antigen-presenting cells, and Th2 and Th17 cell activation; on the other hand, IL-10 promotes B-cell survival, proliferation, differentiation, and antibody production. It is believed to have a dual role in SLE, where the relationship between IL-10 levels and disease activity is conflicting (62–66).

## Role of B1 Cells in Human Systemic Lupus Erythematosus

As mentioned above, CD5 is a marker surface molecule for B1 cells in mice; however, in humans, it does not help to uniquely localize B1 cells. Initially, the CD5 molecule was used as a marker for human B1 cells. An increase in B1 cells that could produce multi-reactive antibodies was observed in patients with a variety of autoimmune diseases, such as rheumatoid arthritis and SLE (40, 67–69). However, it was subsequently found that some pre-naive, transitional, and activating phase B2 cells are also capable of expressing CD5 molecules. Furthermore, some CD5-negative B cells have the characteristic spontaneous antibody-secreting ability of B1 cells (70–75). Therefore, identifying B1 cells in humans is an important initial problem to be solved.

Based on the characteristic functional profile of B1 cells (i.e., spontaneous IgM secretion, stimulation of T-cell activation, and tonic intracellular signaling), Griffin et al. first identified CD20+CD27+CD43+CD70− cells in human umbilical cord blood and peripheral blood as human B1 cells and performed a detailed analysis of the expression of CD5 molecules in this cell type: approximately 75% of these cells expressed CD5 molecules, and the remainder were negative; in contrast, only around one-third of CD5+ cells matched this phenotype, which is consistent with previous reports of partially CD5-expressing B2 cells or CD5− B1 cells (73, 76). Several subsequent studies determined the presence, function, and variation of CD20+CD27+CD43+CD70− cells in human conditions, such as common immune deficiency, multiple sclerosis, and SLE, with different functional profiles (70, 72, 76–78). However, unlike mouse B1 cells, human B1 and B2 cells are derived from the same progenitor lineage: LIN-CD34+CD38lo stem cells (14). Having identified the phenotype of human B1 cells as CD20+CD27+CD43+CD70−, the following question remains: what is their role in SLE?

### IgM Secretion

NIgM are mainly produced spontaneously by B1 cells, have low affinity and multi-reactivity, and act as the first line of immune defense as well as a scavenger of self-apoptotic components (8, 20, 39, 70, 74). It has been found that the presence of anti-phosphatidylcholine, malondialdehyde, and oxidized cardiolipin IgM in SLE patients is protective against cardiovascular disease, especially atherogenesis, *via* the possible mechanisms of increased phagocytosis of apoptotic cells and reduced oxidative stress (7, 79, 80). In addition, this multi-reactive IgM recognizes multiple autoantigens and under certain conditions serves as a template for high-affinity autoantibodies in SLE patients (81). As mentioned previously, a fraction of peripheral B1 cells have high RAG expression in mice, which has also been observed in SLE patients, where a proportion of CD5+ cells had high RAG expression, and this proportion was reduced following cyclophosphamide treatment (81, 82). From this perspective, when B1 cells are altered in SLE patients, such as through migration, class switching, and reduced numbers, they produce less nIgM for clearance of their own components, which results in greater exposure to self-antigen recognition by antigen-presenting cells. On the one hand, this stimulates B1 cells to enhance the expression of RAG and produce different antibodies; on the other hand, this is more likely to activate B2 cells and produce autoimmune antibodies (44).

### Stimulation of CD4+ T Cells

Stimulation of T-cell proliferation, activation, and differentiation is a functional characteristic of B1 cells and is equally important in human B1 cells and animal B1 cells. This function was the target for the identification of the human B1 cell population. Studies later revealed that within the B1 cell population of SLE patients, a predominantly CD11b+ population activates CD4+ T cells, which stimulate the activation and proliferation of CD4+ T cells by upregulating the CD86 molecule, mainly toward the Th17 cell population. In contrast, the CD11b− B1 cells primarily act as antibody secretors (71, 73, 76, 77). However, it is not known whether these B1 cells stimulate T-cell proliferation *in vivo*.

### Other Roles

As mentioned previously, B1 cells are a special type of Breg cells in mice, which secrete IL-10 and regulate immune function. It has been found that numerous CD5+ cells secrete IL-10 in neonates to inhibit inflammation and prevent excessive immune responses (83). Subsequently, an increase in IL-10-secreting CD5+ cells was also found in SLE patients, and Breg cells were mainly derived from innate immune cells, including B1 cells. At the same time, several scholars noted that although the number of Breg cells in SLE patients increases, their capacity to secrete IL-10 decreases (63). Moreover, B1 cells, namely, CD11b+ cells, secrete IL-10 to inhibit T-cell activation *via* the CD3 pathway (34, 77). In addition to secreting cytokines, B1 cells have been reported to exist in the kidneys of patients with lupus nephritis and may interact with other cells to participate in the local immune response.

## Summary

The importance of B cells in SLE is indisputable, and various therapies targeting B cells have been successful to some extent. However, B cells, such as B2 and Breg cells, continue to be a research hotspot in SLE. Although several studies have revealed that B1 cells are involved in the development of SLE by secreting antibodies, activating T cells, producing cytokines, and migrating to target organs, their specific role in human autoimmune diseases requires further exploration. This review summarizes what is known currently about the role of B1 cells in SLE and provides new directions for exploring the pathogenesis and treatment modalities of SLE. Several questions still remain to be explored, such as how to determine the B1 cell phenotype in humans and how B1 cells regulate their own proliferation and secretion. The presence of other cells or factors that can regulate B1 cells is important for developing treatments.

## Author Contributions

ZS prepared the figure. ZS, CL, FW, JM, MH, AA, SL, WW, JT, and CW drafted the manuscript. ZS and CW edited and revised the manuscript. All authors contributed to the article and approved the submitted version.

## Funding

This work was supported by grants from the Natural Science Foundation of Hunan Province in China (grant no. 2019JJ40413), the National Natural Science Foundation of China (grant no. 82070758), and the Hunan Provincial Key R&D Program Project (grant no. 2020SK2084).

## Conflict of Interest

The authors declare that the research was conducted in the absence of any commercial or financial relationships that could be construed as a potential conflict of interest.

## Publisher’s Note

All claims expressed in this article are solely those of the authors and do not necessarily represent those of their affiliated organizations, or those of the publisher, the editors and the reviewers. Any product that may be evaluated in this article, or claim that may be made by its manufacturer, is not guaranteed or endorsed by the publisher.
